# Bioactive small-molecule gas delivery for enhanced bone regeneration: modulation of cellular signaling and remodeling of the regenerative microenvironment

**DOI:** 10.3389/fcell.2026.1992564

**Published:** 2026-10-12

**Authors:** Zhou Zhong, Biao Cao, Xin Duan

**Affiliations:** 1 Department of Orthopedics, West China Hospital, Sichuan University, Chengdu, China; 2 Department of Orthopedics, Orthopedic Research Institute, West China Hospital, Sichuan University, Chengdu, China; 3 Department of Orthopaedics, The Fifth People’s Hospital of Sichuan Province, Chengdu, China

**Keywords:** bone regeneration, controlled release, gas-delivering biomaterials, gasotransmitters, regenerative microenvironment, small-molecule gases, tissue engineering

## Abstract

Complex bone defects are characterized not only by structural loss but also by persistent inflammation, insufficient vascularization, oxidative stress, impaired cell recruitment, and disrupted bone remodeling, which collectively limit endogenous repair. Bioactive small-molecule gases have emerged as versatile regulators of bone regeneration because of their rapid diffusion, low-dose activity, and ability to modulate multiple cellular and microenvironmental processes. This mini-review summarizes the biological roles and biomaterial-based delivery strategies of nitric oxide (NO), carbon monoxide (CO), hydrogen sulfide (H_2_S), oxygen (O_2_), and hydrogen (H_2_) in bone repair. NO primarily promotes angiogenesis and osteogenic–angiogenic coupling, whereas CO contributes mainly to anti-inflammatory, antioxidant, and cytoprotective regulation. H_2_S exhibits broader effects by coordinating redox homeostasis, macrophage polarization, vascularization, mitochondrial function, and osteogenic differentiation. O_2_ and H_2_ provide complementary benefits by alleviating hypoxia, reducing oxidative damage, and remodeling senescence-associated microenvironments. To overcome the rapid diffusion, short half-life, limited local retention, and narrow therapeutic windows of free gases, diverse delivery platforms have been developed, including nanoscale gas-generating systems, stimuli-responsive materials, injectable hydrogels, biomimetic scaffolds, and living gas-producing biomaterials. These systems enable localized, sustained, or on-demand gas release and can integrate immunomodulation, reactive oxygen species scavenging, angiogenic stimulation, bioelectric cues, and mechanical support. Representative studies demonstrate promising outcomes in critical-sized, osteoporotic, diabetic, inflammatory, and aging-related bone defects. Nevertheless, clinical translation remains constrained by challenges in dose control, release kinetics, material degradation, biosafety, long-term metabolism, and stage-specific therapeutic adaptation. Future research should focus on precisely matching gas release profiles to the temporal phases of bone healing and on developing multifunctional platforms that combine gaseous signaling with other regenerative cues.

## 1 Introduction

Bone tissue possesses an intrinsic capacity for self-repair. However, under complex conditions such as extensive trauma, infection, tumor resection, metabolic bone diseases, and aging-related bone injury, local damage often exceeds the endogenous repair capacity, resulting in delayed bone healing, nonunion, or suboptimal regeneration (Bahney et al., 2019; Ehlen et al., 2024). Complex bone defects do not merely represent structural deficiencies; their repair is collectively hindered by persistent inflammation, insufficient vascular reconstruction, elevated oxidative stress, impaired cell recruitment, and an imbalance between bone formation and resorption (Maruyama et al., 2020; ElHawary et al., 2021; Bădilă et al., 2022). Therefore, bone regenerative therapies should extend beyond defect filling and structural replacement to encompass the restoration of local cellular functions and reconstruction of the regenerative microenvironment. Current bone repair strategies primarily rely on bone grafts and artificial substitutes to provide structural support or osteoconductive cues. However, under complex pathological conditions, structural support or biological induction alone is often insufficient to meet the multifaceted requirements of tissue regeneration (Wang and Yeung, 2017; Percival et al., 2024). In recent years, the design paradigm of bone tissue-engineering materials has gradually shifted from passive repair toward active regulation, with increasing emphasis on enhancing bone regeneration through material-mediated signaling modulation, immunomodulation, promotion of vascularization, and microenvironmental remodeling (Dang et al., 2018; Tsukasaki and Takayanagi, 2019). Against this background, bioactive molecules and functional material systems capable of simultaneously regulating cellular behavior and the local microenvironment have attracted considerable attention. Small-molecule gases constitute a distinct class of bioactive signaling mediators that participate in tissue repair by regulating cellular signal transduction, redox homeostasis, inflammatory responses, and angiogenesis (Robert and Subramaniam, 2022; Zafonte et al., 2022). Owing to their small molecular size, high diffusibility, and versatile modes of action, these gaseous molecules exhibit considerable potential for modulating bone regeneration-related cellular behavior and pathological microenvironments (Sun J. et al., 2023). Nevertheless, their direct therapeutic application is limited by poor *in vivo* stability, short local retention, difficulties in controlling release kinetics, and narrow therapeutic windows (Hu et al., 2021; Sarkar et al., 2024). The emergence of bioactive small-molecule gas-delivering biomaterials provides a promising strategy for overcoming these limitations. Through the loading, *in situ* generation, or stimulus-responsive release of gaseous molecules, biomaterial-based systems can achieve more stable, localized, and controllable gas delivery within bone defects, thereby promoting osteogenesis, vascularization, immune homeostasis, and microenvironmental reconstruction (Hu et al., 2021; Sun J. et al., 2023; Sarkar et al., 2024). Compared with conventional bone repair materials, these systems place greater emphasis on the dynamic regulation of the entire bone regenerative process, reflecting a conceptual shift from structural repair alone toward multidimensional biological modulation.

Accordingly, this mini-review systematically summarizes recent advances in bioactive small-molecule gas-delivering biomaterials for bone regeneration. It discusses the regulatory roles of gaseous signaling molecules in cellular processes, outlines common strategies for constructing gas-generating and gas-delivering biomaterials, and highlights their functional effects and mechanisms of microenvironmental modulation during bone regeneration. This review aims to provide a theoretical basis and research framework for the rational design of bioactive small-molecule gas-delivering biomaterials, the elucidation of their underlying mechanisms, and their translational application in bone regeneration and repair.

## 2 Gasotransmitters and the regulation of cellular signaling in bone regeneration

Throughout the aforementioned stages of bone repair, cellular signaling networks determine the coordination among inflammation resolution, angiogenesis, stem cell differentiation, and bone remodeling. Any disruption in these interconnected processes may compromise the rate and quality of new bone formation (Grosso et al., 2017; ElHawary et al., 2021; Wang et al., 2025). Owing to their small molecular size, high diffusibility, ability to cross cell membranes, and diverse molecular targets, gasotransmitters can rapidly modulate the behavior of multiple cell types involved in bone regeneration within the local microenvironment. Nitric oxide (NO), carbon monoxide (CO), and hydrogen sulfide (H_2_S) are the three most extensively studied endogenous gasotransmitters. By regulating vascular responses, inflammatory processes, antioxidant defenses, mitochondrial homeostasis, and cell fate decisions, these molecules exert broad effects on cellular networks associated with bone regeneration (Figure 1) (Sarkar et al., 2024). Therefore, elucidating the actions of these gaseous molecules from the perspective of cellular signaling is essential for understanding the biological rationale underlying their incorporation into bone repair materials.

**FIGURE 1:**
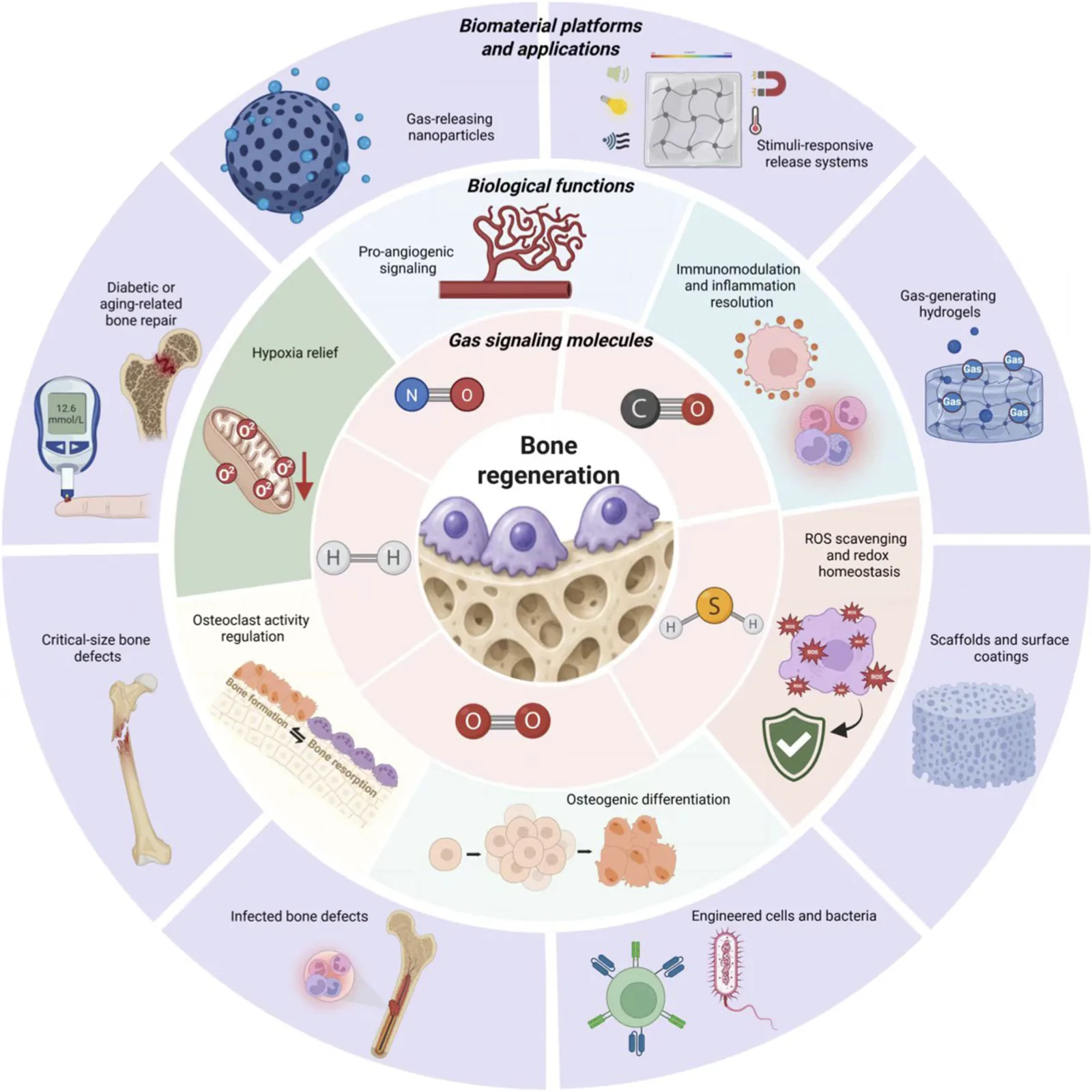
Schematic overview of bioactive small-molecule gas-delivering biomaterials and their regulatory functions in bone regeneration.

### 2.1 Biological characteristics of classical gasotransmitters

NO, CO, and H_2_S are endogenously generated through specific enzymatic reactions and function as signaling mediators under physiological conditions or within relatively low concentration ranges (Sun J. et al., 2023). Unlike most protein-based factors, these gaseous molecules do not depend exclusively on recognition by cell-surface receptors. Instead, they can diffuse directly into cells and subcellular organelles, where they interact with metalloproteins, thiol-containing proteins, second-messenger systems, and redox-sensitive molecular targets (Sarkar et al., 2024). A shared feature of these gasotransmitters is that their biological effects are highly dependent on local concentration, exposure duration, and cellular context. At appropriate levels, they contribute to tissue homeostasis and injury repair; however, sustained elevation or uncontrolled release may induce nitrosative stress, mitochondrial inhibition, metabolic dysfunction, and even cellular injury (Sun J. et al., 2023; Sarkar et al., 2024). Accordingly, the application of gasotransmitters in bone regeneration should emphasize their therapeutic concentration windows, release kinetics, and microenvironment-dependent effects rather than regarding them simply as unidirectional pro-regenerative factors. Although representative concentration ranges can be derived from individual experimental studies, a universal therapeutic window has not yet been established for these gasotransmitters because their biological effects are strongly influenced by donor chemistry, release kinetics, exposure duration, and the local microenvironment.

### 2.2 NO: a dose-dependent regulator of angiogenesis and osteogenesis

NO is primarily generated from L-arginine through reactions catalyzed by nitric oxide synthases and serves as an important signaling molecule linking vascular function to bone tissue repair. At appropriate concentrations, NO activates the soluble guanylate cyclase/cyclic guanosine monophosphate (sGC/cGMP) signaling axis, thereby promoting vasodilation, endothelial cell migration, and neovascularization. These effects improve perfusion within bone defects and facilitate the recruitment of reparative cells and the deposition of new bone matrix (Cheng et al., 2024). NO can also regulate the functional states of mesenchymal stem cells, osteoblasts, and immune cells, contributing to osteogenic–angiogenic coupling and the modulation of early inflammatory responses (Grosso et al., 2017; Won et al., 2022). Nevertheless, the biological effects of NO are distinctly biphasic. Under conditions of persistent inflammation, excessive activation of inducible nitric oxide synthase may lead to the overproduction of NO. NO can subsequently react with reactive oxygen species (ROS) to generate reactive nitrogen species, resulting in mitochondrial damage, impaired cellular activity, and disrupted bone formation (Sun J. et al., 2023). Therefore, the central objective of NO-based strategies for bone regeneration is not to maximize the total amount of NO released, but rather to maintain its concentration within a therapeutically effective range that supports vascularization and osteogenesis.

Representative *in vitro* studies further illustrate the dose- and release kinetics-dependent effects of NO on osteoblasts. In primary rat osteoblasts, the slow-releasing NO donor NOC-18 at 10–50 μM promoted cell proliferation and alkaline phosphatase activity, whereas the rapidly releasing donor sodium nitroprusside (SNP), particularly at 100 μM, inhibited proliferation and induced apoptosis (Mancini et al., 2000). Consistently, continuous treatment with 10 μM NOC-18 enhanced osteocalcin expression and increased the formation of mineralized nodules in rat osteoblastic cells (Otsuka et al., 1998). More recent evidence has also shown that low-dose NO-donor treatment can enhance osteogenic activity, with NOC22 at 0.1–10 μM increasing alkaline phosphatase activity and promoting osteoblast-associated bone formation (Zhao et al., 2018). These findings indicate that the therapeutic window of NO cannot be defined solely by nominal donor concentration, because its biological effects are also strongly influenced by donor chemistry, release rate, cumulative exposure, and treatment duration. Therefore, NO-releasing biomaterials should prioritize sustained and controlled low-level delivery while avoiding rapid or excessive local NO accumulation.

### 2.3 CO: an anti-inflammatory, antioxidant, and cytoprotective gasotransmitter

CO is predominantly produced through heme degradation mediated by heme oxygenases, among which the heme oxygenase-1 (HO-1)/CO system represents an important cytoprotective mechanism against cellular stress (Ryter, 2019). At low concentrations, CO can suppress excessive inflammatory responses, attenuate oxidative damage, and improve mitochondrial function and cell survival. In the context of complex bone defects, CO appears to act primarily by ameliorating inflammatory and oxidative microenvironments rather than functioning as a potent osteoinductive signal on its own. Previous studies have indicated that CO-releasing molecules can promote the osteogenic differentiation of periodontal ligament stem cells and bone marrow-derived mesenchymal stem cells, potentially through the regulation of osteogenesis-associated pathways such as Wnt signaling (Chen et al., 2021; Li et al., 2022). Thus, CO may be more appropriately regarded as a gasotransmitter that supports bone formation by providing cytoprotection and restoring the local microenvironment, while synergistically enhancing osteogenesis under specific conditions.

CO also exhibits a concentration-dependent biphasic effect. In human periodontal ligament stem cells, CORM-3 at 100, 200, and 400 μM showed a tendency to promote cell proliferation, with a significant effect observed at 400 μM, whereas 800 μM CORM-3 significantly reduced cell viability (Chen et al., 2021). In rat bone marrow mesenchymal stem cells, 200 μM CORM-3 has also been shown to enhance osteogenic differentiation and mineralization (Li et al., 2022). However, these concentrations represent the nominal concentrations of CO-releasing molecules rather than the actual concentrations of bioavailable CO. Moreover, the amount and kinetics of CO release vary among CORM systems, and accumulating evidence indicates that some CORMs, including CORM-3, may exhibit CO-independent chemical and biological activities (Bauer et al., 2023). Therefore, the effective dose of CO cannot be defined solely on the basis of donor concentration but should also consider CO-release efficiency, release kinetics, exposure duration, and local tissue accumulation.

### 2.4 H_2_S: a gasotransmitter linking antioxidant defense, angiogenesis, and osteogenesis

H_2_S is endogenously generated by enzyme systems involved in the metabolism of sulfur-containing amino acids and has attracted increasing attention in the field of bone regeneration in recent years (Gambari et al., 2019b; Cirino et al., 2023). Compared with NO and CO, H_2_S exhibits a broader spectrum of regulatory activities. It can attenuate oxidative damage in pathological bone defects by activating nuclear factor erythroid 2-related factor 2 (Nrf2)-associated antioxidant responses, reducing ROS accumulation, and preserving mitochondrial function. In parallel, H_2_S can modulate signaling networks involving vascular endothelial growth factor (VEGF), phosphoinositide 3-kinase/protein kinase B (PI3K/Akt), mitogen-activated protein kinase (MAPK), and Wnt/β-catenin, thereby enhancing endothelial cell function, angiogenesis, and osteogenic lineage differentiation (Cirino et al., 2023). In addition, H_2_S can regulate macrophage phenotypic polarization, which may help mitigate the detrimental effects of persistent inflammation on bone repair (Zhou et al., 2023; Zhou Y. K. et al., 2024). On the basis of these properties, H_2_S may be considered a multifunctional gasotransmitter with antioxidant, pro-angiogenic, immunomodulatory, and osteogenic potential.

### 2.5 Crosstalk among NO, CO, and H_2_S

NO, CO, and H_2_S do not function independently. Instead, they engage in complex crosstalk through shared regulatory nodes, including heme-containing proteins, thiol modifications, second-messenger systems, and mitochondrial metabolism (Oza and Kashfi, 2023; Zhou Y. K. et al., 2024). These gasotransmitters exhibit distinct but overlapping roles in bone regeneration (Figure 2). NO is more closely associated with vasodilation and osteogenic–angiogenic coupling; CO primarily contributes to HO-1-related cytoprotection and suppression of inflammation; and H_2_S exerts broader effects on antioxidant defense, immune-cell phenotypic regulation, angiogenesis, and osteogenesis (Gambari et al., 2019b; Ryter, 2019; Sun J. et al., 2023). These observations suggest that future gas-delivery strategies should not be limited to evaluating the effects of individual gases. Greater attention should also be given to the relative doses of different gasotransmitters, their sequence of release, and their compatibility with the stage-specific requirements of bone repair.

**FIGURE 2:**
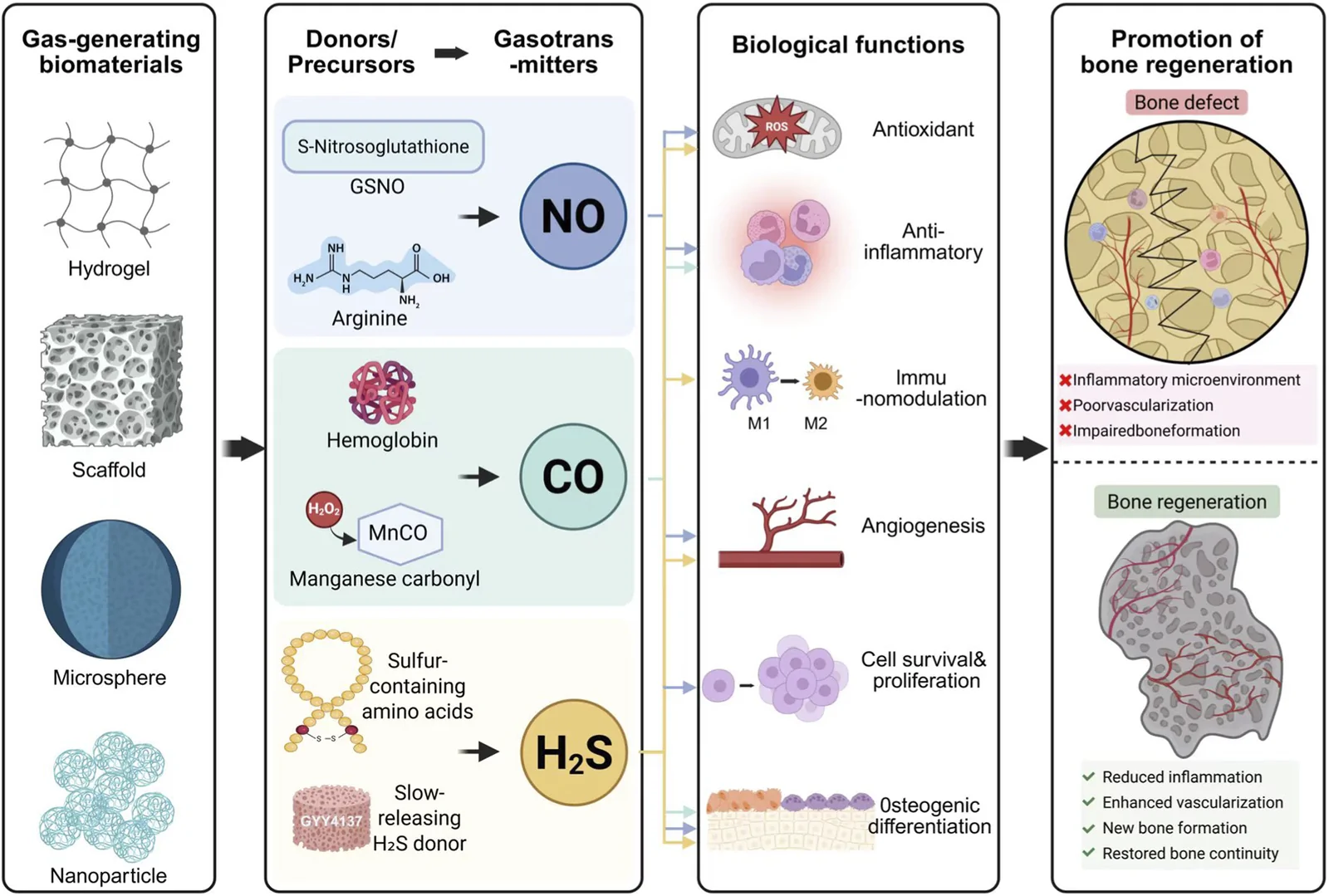
Gasotransmitter-releasing biomaterials for enhanced bone regeneration: from gas donor incorporation to biological regulation and tissue repair.

## 3 Functional gas delivery and gas-generating biomaterials

Having established the regulatory effects of gasotransmitters such as NO, CO, and H_2_S on the behavior of cells involved in bone regeneration, the stable generation, controlled release, and effective utilization of these gases at bone defect sites have become critical to the clinical translation of gas therapy. Free gaseous molecules are generally characterized by rapid diffusion, short half-lives, difficulty in maintaining effective local concentrations, and narrow therapeutic windows. Consequently, direct exogenous administration is often unable to meet the spatiotemporal regulatory requirements of bone regeneration (Hu et al., 2021; Sun J. et al., 2023). Therefore, the design of gas-generating biomaterials should extend beyond the simple release of gaseous molecules. Instead, material architecture, reaction mechanisms, and microenvironment-responsive properties should be coordinated to align gas generation with the stage-specific requirements of bone defect repair, thereby enabling the integrated regulation of inflammation, oxidative stress, angiogenesis, and osteogenic differentiation (Sarkar et al., 2024).

### 3.1 Nanoscale gas-generating materials

Nanoscale gas-generating materials represent one of the most widely investigated platforms for gas delivery and mainly include inorganic nanoparticles, mesoporous nanocarriers, nanoreactors, and nano-/micromotors. These materials possess a large specific surface area, readily modifiable surfaces, high reaction activity, and tunable tissue distribution, making them suitable for loading gas donors, catalyzing *in situ* gas generation, and regulating gas-release kinetics (Hu et al., 2021; Wang et al., 2023; Hamza et al., 2025). Inorganic nanomaterials can exploit their catalytic, degradative, or redox properties to generate O_2_, NO, H_2_S, or other therapeutic gases within the bone defect microenvironment. Mesoporous nanomaterials, by contrast, rely on their ordered pore structures and high loading capacities to stably encapsulate gas donors and achieve sustained release. Nanomotors and micro-/nanoscale propulsion systems can generate local motion through gas-producing reactions, thereby enhancing material penetration into complex lesions or biofilm-associated environments; however, their direct application in bone defect repair requires further validation (Wan et al., 2019; Zheng et al., 2022; Wang et al., 2023). In the context of bone regeneration, nanoscale gas-generating materials offer the advantage of integrating gas production, localized delivery, and microenvironmental modulation within a single platform. Nevertheless, their long-term metabolism, tissue accumulation, dose controllability, and potential toxicity remain important issues that require systematic evaluation (Hu et al., 2021).

### 3.2 Stimuli-responsive gas-generating materials

Stimuli-responsive gas-generating materials are designed to achieve on-demand gas release in response to pathological features within bone defects or externally applied physical stimuli. Complex bone defects are frequently accompanied by local acidosis, elevated ROS levels, abnormal enzymatic activity, hypoxia, and the accumulation of inflammatory mediators. These aberrant signals can serve as triggers for gas release, allowing gas generation to adapt dynamically to changes in the pathological microenvironment (Ji and Zhong, 2022; Rong et al., 2023). For example, pH-responsive systems can accelerate the decomposition of gas donors within acidic inflammatory environments. ROS-responsive systems can release anti-inflammatory or antioxidant gases in parallel with increases in oxidative stress, whereas enzyme-responsive systems can regulate release through the activity of specific enzymes. In addition, external stimuli, including light, ultrasound, magnetic fields, and temperature, can be used to remotely control gas release, thereby improving the temporal precision and spatial selectivity of treatment (Cheng et al., 2024). Compared with continuously and passively releasing systems, stimuli-responsive gas-generating materials are more effective in minimizing ineffective release and excessive exposure. However, their design must balance response sensitivity, release stability, biosafety, and the efficient transmission of triggering signals into deep bone defect tissues.

In bone regeneration, spatial specificity is primarily achieved through local implantation or injection of gas-generating hydrogels and scaffolds directly into bone defects, thereby enhancing local retention and reducing unnecessary systemic exposure (Suvarnapathaki et al., 2022; Sarkar et al., 2024). In addition, microenvironment-responsive systems can provide temporal control by coupling therapeutic release to pathological signals associated with bone defects, including acidic pH, elevated ROS levels, and abnormal enzymatic activity (Wei et al., 2022; Rong et al., 2023). Such responsiveness provides a potential means of adapting gas delivery to the evolving local microenvironment during tissue repair, thereby reducing unnecessary or excessive exposure. External stimuli, including light, ultrasound, and magnetic fields, may further improve temporal control over gas release (Ji and Zhong, 2022; Cheng et al., 2024). Thus, combining local defect retention with microenvironment-responsive or externally triggered release represents a practical strategy for achieving spatially localized and temporally regulated gas delivery during bone regeneration.

### 3.3 Gas-generating hydrogels

Gas-generating hydrogels combine a three-dimensional network structure, high water content, favorable biocompatibility, and injectability, making them particularly suitable for the local filling and sustained treatment of irregularly shaped or deep bone defects (Sarkar et al., 2024). Gas donors, catalytic components, or gas-generating particles can be incorporated into hydrogels through physical encapsulation, chemical conjugation, dynamic crosslinking, or nanocomposite construction. These approaches create a localized reactive microenvironment within bone defects and enable sustained gas release, *in situ* gas generation, or stimuli-responsive delivery (Suvarnapathaki et al., 2022). Compared with particulate carriers, hydrogels more effectively maintain local retention and reduce fluctuations in gas concentration caused by rapid diffusion. They can also provide an extracellular matrix-like microenvironment that supports cell adhesion, migration, and matrix deposition. In bone regeneration, gas-generating hydrogels can regulate inflammation, oxidative stress, and vascularization through the release of NO, CO, H_2_S, O_2_, or H_2_. They can also be integrated with inorganic mineral components, growth factors, therapeutic agents, or extracellular vesicles to construct multifunctional regenerative platforms (Chen et al., 2023; Fang et al., 2024). Nevertheless, hydrogel-based systems still face several limitations, including insufficient mechanical strength, difficulty in maintaining stable long-term gas-release kinetics, and a mismatch between material degradation and new bone formation.

### 3.4 Living gas-generating biomaterials: engineered cells, engineered bacteria, and related systems

Living gas-generating biomaterials are an emerging class of gas-producing platforms with intrinsic biological activity and the potential for adaptive regulation. These systems mainly include engineered cells, engineered bacteria, microalgae, and other living entities capable of producing therapeutic gases (Pan et al., 2026). Unlike conventional materials, living gas-generating systems can exploit their endogenous metabolic activity to continuously produce specific gases or modulate the local microenvironment. They may also respond dynamically to changes in nutrient availability, oxygen tension, inflammatory status, or metabolic signals. Engineered cells can be designed as local release units for therapeutic gases or cytoprotective factors while simultaneously contributing to immunomodulation, vascularization, and tissue repair. Engineered bacteria and photosynthetic microorganisms can generate NO, H_2_S, O_2_, or other bioactive small molecules through metabolic reactions, thereby providing new therapeutic approaches for ischemic, hypoxic, or inflammatory bone defects (Wang et al., 2022; Hayes et al., 2023; Ortega et al., 2023). However, the application of living biomaterials in bone regeneration remains associated with substantial translational challenges, including biosafety, immunogenicity, genetic stability, *in vivo* persistence, controllable elimination, and potential infection risks. Accordingly, these systems are more appropriately regarded as a future direction for intelligent and controllable gas-therapeutic platforms and require systematic validation of both safety and long-term therapeutic efficacy.

## 4 Roles of gas-generating biomaterials in bone regeneration

In bone regeneration applications, the focus of gas-generating biomaterials has gradually shifted from the simple delivery of gaseous molecules toward functional interventions tailored to specific pathological microenvironments. Different gas-based materials exhibit distinct advantages in promoting vascularization, modulating immune responses, alleviating oxidative stress, and inducing osteogenesis. Therefore, their bone-regenerative effects should be further summarized according to the type of gas involved (Sun J. et al., 2023; Sarkar et al., 2024).

### 4.1 NO-generating biomaterials for bone regeneration

Among currently available gas-generating materials, NO-releasing systems are supported by relatively extensive evidence in the field of bone regeneration. Their principal therapeutic effects involve enhancing vascularization and regulating the osteogenic–immune microenvironment to facilitate bone repair. Won et al. developed a polymeric system capable of controlled NO release for guided bone regeneration. The NO-releasing polymer enhanced alkaline phosphatase (ALP) activity and mineralization in preosteoblasts while promoting vascularization and new bone formation in a critical-sized calvarial defect model (Won et al., 2022). Lee et al. subsequently designed a biomimetic NO-releasing scaffold for osteoporotic bone regeneration. By integrating NO-releasing capacity into a bone-mimetic poly (lactic-co-glycolic acid) (PLGA)-based composite structure, the scaffold showed advantages in restoring bone homeostasis, promoting osteogenesis, and suppressing pathological bone resorption (Lee et al., 2023). In addition, the adaptive biomimetic periosteum reported by Zhou et al. enhanced angiogenesis within bone defects through NO release, suggesting that NO-based materials may be particularly suitable for repairing bone defects characterized by insufficient vascularization (Zhou Z. et al., 2024). More recently, Cheng et al. developed a photothermally responsive NO-releasing system that further highlighted the importance of spatiotemporal control over NO delivery. External stimulation accelerated NO release from this system, thereby enhancing bone repair through the simultaneous promotion of osteogenesis and angiogenesis (Cheng et al., 2024). Collectively, these studies demonstrate that the key to applying NO-based materials in bone regeneration is not simply to increase the total amount of NO released, but rather to maintain NO within an effective therapeutic window that supports angiogenesis, cell recruitment, and osteogenic differentiation through controlled delivery.

### 4.2 H_2_S-generating biomaterials for bone regeneration

The bone-regenerative effects of H_2_S-generating materials are primarily associated with antioxidant activity, immunomodulation, enhanced vascularization, and promotion of osteogenesis. Gambari et al. (2019a) developed an H_2_S-releasing silk fibroin scaffold that supported cellular behaviors relevant to bone tissue engineering *in vitro*, indicating that H_2_S may serve as a bioactive regulatory component in bone repair scaffolds. In a more complex pathological bone defect model, Jiang et al. designed an injectable ROS-responsive H_2_S-releasing hydrogel. This system consisted of an alginate network containing S-aroylthiooxime (SATO)-based H_2_S-generating groups and a poly (ethylene glycol) diacrylate (PEGDA) network, together with ROS-responsive cysteine-releasing microparticles. The hydrogel scavenged excessive ROS, promoted the osteogenic differentiation of bone marrow-derived mesenchymal stem cells (BMSCs), regulated macrophage polarization, suppressed inflammation and apoptosis, and enhanced bone repair in an osteoporotic bone defect model (Jiang et al., 2024). Fang et al. reported a polyphenol-mediated redox-active hydrogel that combined sustained H_2_S release with a conductive microfiber network for diabetic periodontal bone healing. In this system, bovine serum albumin nanoparticles enabled sustained H_2_S release, which acted synergistically with the conductive architecture, antioxidant activity, and immunomodulatory functions to promote mesenchymal stem cell recruitment, angiogenesis, and osteogenesis (Fang et al., 2024). These findings suggest that H_2_S-releasing systems are particularly suitable for pathological bone defects characterized by pronounced oxidative stress and immune dysregulation, as they can simultaneously restore reparative cell function and ameliorate the local inflammatory microenvironment.

### 4.3 CO-releasing systems and related biomaterials for bone regeneration

Compared with NO- and H_2_S-releasing systems, biomaterial-based evidence supporting the application of CO in bone defect repair remains relatively limited. Current studies have focused primarily on the effects of CO-releasing molecules on osteogenic differentiation and the regulation of inflammatory bone loss by nanoscale CO-delivery systems. Chen et al. demonstrated that the CO-releasing molecule CORM-3 promoted the osteogenic differentiation of human periodontal ligament stem cells (hPDLSCs) and enhanced hPDLSC-mediated new bone formation in a critical-sized calvarial defect model (Chen et al., 2021). Li et al. (2022) further found that CORM-3 promoted the osteogenic differentiation of rat bone marrow-derived mesenchymal stem cells through a miR-195–5p/Wnt3a-associated pathway, suggesting that CO may contribute to bone formation by modulating Wnt-related osteogenic signaling. With respect to biomaterial-mediated delivery, Liu et al. (2025) developed a CO@MPN nanocomposite in which CORM-3 was encapsulated within a metal–phenolic network. This platform stabilized CO-releasing molecules and enabled ROS-responsive CO release. In an experimental periodontitis model, CO@MPN reduced ROS levels, suppressed the release of pro-inflammatory mediators, promoted macrophage polarization toward the M2 phenotype, and markedly alleviated inflammatory bone loss. Accordingly, the role of CO-generating materials in bone regeneration should be positioned primarily as the regulation of inflammatory and oxidative-stress microenvironments and the support of osteogenic potential, rather than being directly equated with strongly osteoinductive scaffolds. In the future, integrating controlled CO release with osteoconductive scaffolds, hydrogels, or membrane-based materials may further enhance its therapeutic value in infectious, inflammatory, or metabolically compromised bone defects.

### 4.4 Integration of gaseous signaling with other therapeutic modalities for bone regeneration

To date, relatively few studies have directly investigated the combined release of two or more classical gasotransmitters for bone regeneration. In contrast, multifunctional systems combining gas release with antioxidant activity, electrical conductivity, therapeutic ion release, oxygen supply, or anti-senescence functions have been more frequently reported. The H_2_S gas–bioelectric coupling hydrogel developed by Fang et al. represents a typical example. Rather than merely releasing H_2_S, this system integrated sustained H_2_S delivery, conductive microfibers, ROS scavenging, and immunomodulation within a single platform. By promoting cellular alignment, calcium influx, autophagic activity, and macrophage polarization, the hydrogel ameliorated the diabetic periodontitis microenvironment and enhanced periodontal bone regeneration (Fang et al., 2024). The CPP-L/GelMA hydrogel constructed by Sun et al. represents an antioxidant–oxygen-supply synergistic strategy. This system simultaneously incorporated catalase and ROS-responsive oxygen-releasing nanoparticles, enabling ROS scavenging and prolonged oxygen supply according to the local requirements of the defect. In a murine calvarial defect model, the hydrogel promoted angiogenesis and osteogenesis while suppressing osteoclast-related processes (Sun H. et al., 2023). The localized H_2_-releasing system reported by Chen et al. targeted the aging-associated microenvironment. By locally releasing hydrogen to attenuate inflammation and cellular senescence, this system improved bone defect repair in aged animals (Chen et al., 2023). These studies indicate that the future development of synergistic gas-based biomaterials should not be restricted to the simultaneous release of multiple gases. Instead, multifunctional regenerative platforms should be rationally designed according to the specific pathological characteristics of bone defects, integrating gaseous signaling with immunomodulation, oxidative stress control, angiogenic stimulation, ion-mediated regulation, and mechanical support.

## 5 Conclusion

In summary, bioactive small-molecule gases provide an alternative approach to regulating bone regeneration that differs from conventional growth factor- and drug-delivery strategies (Table 1). NO, CO, and H_2_S participate in bone repair by modulating vascular responses, inflammation resolution, oxidative stress, mitochondrial function, and the behavior of osteogenesis-related cells. By contrast, O_2_ and H_2_ mainly exert complementary effects by alleviating hypoxia, enhancing antioxidant defenses, and remodeling aging-associated microenvironments (Hu et al., 2021; Sarkar et al., 2024). Given the pronounced stage dependence and microenvironmental sensitivity of bone regeneration, the transient activity, efficacy at low doses, and multitarget effects of gaseous molecules are well suited to the requirements of dynamic local regulation (ElHawary et al., 2021). Advances in nanocarriers, hydrogels, biomimetic scaffolds, stimuli-responsive systems, and multifunctional gas-generating platforms have further improved local gas retention, on-demand release, and functional integration. Consequently, gas-generating biomaterials are gradually evolving from simple delivery vehicles into functional platforms capable of actively remodeling the regenerative microenvironment (Hu et al., 2021; Won et al., 2022; Fang et al., 2024; Sarkar et al., 2024).

**TABLE 1:** Representative studies of functional small-molecule gas-delivering biomaterials for bone regeneration. The table summarizes representative studies discussed in the mini-review. For studies without *in vivo* animal experiments, the model is indicated as *in vitro*.

Gas molecule	Biomaterial/delivery system	Gas release or responsive strategy	Main mechanism	Animal/experimental model	Major findings	References	Year
NO	NO-releasing polymer system	Controlled NO release	Promotes angiogenesis and osteogenic differentiation; enhances bone-vascular coupling	Critical-sized bone defect model	Enhanced ALP activity, mineralization, vascular formation, and new bone formation	Won et al.; DOI: 10.1002/mabi.202200162	2022
NO	NO-releasing bioinspired scaffold	Scaffold-mediated local NO release	Regulates bone homeostasis; promotes osteogenesis and suppresses abnormal bone resorption	Osteoporotic bone regeneration model	Improved new bone regeneration under osteoporotic conditions	Lee et al.; DOI: 10.1002/advs.202205336	2023
NO	Self-adaptive biomimetic periosteum	Local NO release from a biomimetic periosteum	Augments angiogenesis and improves the regenerative microenvironment in bone defects	Critical-size bone defect regeneration model	Enhanced vascularization and new bone formation during bone defect repair	Zhou et al.; DOI: 10.1002/adhm.202302153	2024
NO	Photothermal-responsive NO-releasing system	Photothermal-triggered NO release	Promotes osteogenesis and angiogenesis through spatiotemporally controlled NO release	Bone defect model	Accelerated NO release and enhanced bone repair	Cheng et al.; DOI: 10.1016/j.mtbio.2024.101180	2024
H_2_S	H2S-releasing silk fibroin scaffold	Sustained H2S release	Supports bone tissue engineering-related cell behavior	*In vitro* bone tissue engineering model	Suggested that H2S can act as a bioactive regulatory cue in bone repair scaffolds	Gambari et al.; DOI: 10.1016/j.msec.2019.04.039	2019
H_2_S	ROS-responsive injectable hydrogel	ROS-triggered H2S release	Scavenges ROS, promotes BMSC osteogenesis, regulates macrophage polarization, and balances osteoclastogenesis	Osteoporotic bone defect model	Reduced oxidative stress and inflammation and enhanced osteoporotic bone repair	Jiang et al.; DOI: 10.1016/j.mtbio.2024.101338	2024
H_2_S	Polyphenol-mediated redox-active hydrogel	Sustained H2S release combined with bioelectric cues	Integrates antioxidation, immunomodulation, cell recruitment, angiogenesis, and osteogenesis	Diabetic periodontal bone defect model	Improved periodontal bone healing under diabetic conditions	Fang et al.; DOI: 10.1038/s41467-024-53290-6	2024
CO	CORM-3-based CO-releasing system	CO release from CORM-3	Promotes osteogenic differentiation of hPDLSCs and supports new bone formation	Critical-sized cranial defect model	Enhanced hPDLSC-mediated new bone formation	Chen et al.; DOI: 10.2147/DDDT.S300356	2021
CO	CORM-3 treatment for BMSCs	CO donor-mediated release	Promotes osteogenic differentiation through the miR-195–5p/Wnt3a-related pathway	*In vitro* rat BMSC model	Enhanced osteogenic differentiation of rat BMSCs	Li et al.; DOI: 10.2147/DDDT.S367277	2022
CO	CORM-3-loaded metal-phenolic network	ROS-responsive CO release	Reduces ROS and inflammatory cytokines and promotes macrophage M2 polarization	Experimental periodontitis model	Alleviated inflammation-associated bone loss	Liu et al.; DOI: 10.1016/j.mtbio.2025.102213	2025
O_2_	CPP-L/GelMA oxygen-generating hydrogel	ROS scavenging and prolonged oxygen generation	Scavenges ROS, relieves hypoxia, promotes angiogenesis and osteogenesis, and suppresses osteoclast-related processes	Mouse calvarial defect model	Promoted vascularized bone formation and improved the bone microenvironment	Sun et al.; DOI: 10.1016/j.bioactmat.2022.12.021	2023
H_2_	Local H2-releasing system	Local hydrogen release	Reduces inflammation and cellular senescence and remodels the senescence microenvironment	Aged bone defect model	Improved repair of injured bone in an aging-related microenvironment	Chen et al.; DOI: 10.1038/s41467-023-43618-z	2023

Abbreviations: ALP, alkaline phosphatase; BMSC, bone marrow mesenchymal stem cell; CO, carbon monoxide; CORM-3, carbon monoxide-releasing molecule-3; CPP-L/GelMA, catalase/PFC@PLGA/PPS, liposome-loaded GelMA, hydrogel; H2, hydrogen; H2S, hydrogen sulfide; hPDLSC, human periodontal ligament stem cell; NO, nitric oxide; O2, oxygen; ROS, reactive oxygen species.

Future gas-delivering biomaterials should emphasize stage-specific and controlled release throughout bone repair, while integrating gaseous signaling with other therapeutic modalities to address complex defects. Beyond general biomaterial-related issues, gas therapy faces additional translational barriers arising from the rapid diffusion and short lifetime of gaseous molecules, including difficulty in real-time monitoring of local gas exposure, challenges in maintaining reproducible release kinetics during large-scale manufacturing, and regulatory uncertainty for systems combining structural and pharmacological functions. Standardized gas-release characterization, manufacturing quality control, and regulatory evaluation will therefore be essential for clinical translation.

## References

[B1] Bădilă A. E. Rădulescu D. M. Ilie A. Niculescu A. G. Grumezescu A. M. Rădulescu A. R. (2022). Bone regeneration and oxidative stress: an updated overview. Antioxidants (Basel) 11 (2), 318. 10.3390/antiox11020318 35204201 PMC8868092

[B2] Bahney C. S. Zondervan R. L. Allison P. Theologis A. Ashley J. W. Ahn J. (2019). Cellular biology of fracture healing. J. Orthop. Res. 37 (1), 35–50. 10.1002/jor.24170 30370699 PMC6542569

[B3] Bauer N. Yuan Z. Yang X. Wang B. (2023). Plight of CORMs: the unreliability of four commercially available CO-releasing molecules, CORM-2, CORM-3, CORM-A1, and CORM-401, in studying CO biology. Biochem. Pharmacol. 214, 115642. 10.1016/j.bcp.2023.115642 37321416 PMC10529722

[B4] Chen H. Dai Y. Cui J. Yin X. Feng W. Lv M. (2021). Carbon monoxide releasing Molecule-3 enhances osteogenic differentiation of human periodontal ligament stem cells by carbon monoxide release. Drug Des. Devel Ther. 15, 1691–1704. 10.2147/dddt.S300356 33911854 PMC8075314

[B5] Chen S. Yu Y. Xie S. Liang D. Shi W. Chen S. (2023). Local H(2) release remodels senescence microenvironment for improved repair of injured bone. Nat. Commun. 14 (1), 7783. 10.1038/s41467-023-43618-z 38012166 PMC10682449

[B6] Cheng Y. Huo Y. Yu Y. Duan P. Dong X. Yu Z. (2024). A photothermal responsive system accelerating nitric oxide release to enhance bone repair by promoting osteogenesis and angiogenesis. Mater Today Bio 28, 101180. 10.1016/j.mtbio.2024.101180 39221216 PMC11364911

[B7] Cirino G. Szabo C. Papapetropoulos A. (2023). Physiological roles of hydrogen sulfide in mammalian cells, tissues, and organs. Physiol. Rev. 103 (1), 31–276. 10.1152/physrev.00028.2021 35435014

[B8] Dang M. Saunders L. Niu X. Fan Y. Ma P. X. (2018). Biomimetic delivery of signals for bone tissue engineering. Bone Res. 6, 25. 10.1038/s41413-018-0025-8 30181921 PMC6115422

[B9] Ehlen Q. T. Costello J. P. Mirsky N. A. Slavin B. V. Parra M. Ptashnik A. (2024). Treatment of bone defects and nonunion via novel delivery mechanisms, growth factors, and stem cells: a review. ACS Biomater. Sci. Eng. 10 (12), 7314–7336. 10.1021/acsbiomaterials.4c01279 39527574 PMC11632667

[B10] ElHawary H. Baradaran A. Abi-Rafeh J. Vorstenbosch J. Xu L. Efanov J. I. (2021). Bone healing and inflammation: principles of fracture and repair. Semin. Plast. Surg. 35 (3), 198–203. 10.1055/s-0041-1732334 34526868 PMC8432998

[B11] Fang X. Wang J. Ye C. Lin J. Ran J. Jia Z. (2024). Polyphenol-mediated redox-active hydrogel with H(2)S gaseous-bioelectric coupling for periodontal bone healing in diabetes. Nat. Commun. 15 (1), 9071. 10.1038/s41467-024-53290-6 39433776 PMC11494015

[B12] Gambari L. Amore E. Raggio R. Bonani W. Barone M. Lisignoli G. (2019a). Hydrogen sulfide-releasing silk fibroin scaffold for bone tissue engineering. Mater Sci. Eng. C Mater Biol. Appl. 102, 471–482. 10.1016/j.msec.2019.04.039 31147018

[B13] Gambari L. Grigolo B. Grassi F. (2019b). Hydrogen sulfide in bone tissue regeneration and repair: state of the art and new perspectives. Int. J. Mol. Sci. 20 (20). 10.3390/ijms20205231 31652532 PMC6834365

[B14] Grosso A. Burger M. G. Lunger A. Schaefer D. J. Banfi A. Di Maggio N. (2017). It takes two to tango: coupling of angiogenesis and osteogenesis for bone regeneration. Front. Bioeng. Biotechnol. 5, 68. 10.3389/fbioe.2017.00068 29164110 PMC5675838

[B15] Hamza H. M. Malik M. Asad M. Ali S. Awan A. (2025). Advances in orthopedic implants: the role of nanotechnology in enhancing performance and longevity. Regen. Med. Rep. 2, 15–21. 10.4103/REGENMED.REGENMED-D-24-00024

[B16] Hayes J. A. Lunger A. W. Sharma A. S. Fernez M. T. Carrier R. L. Koppes A. N. (2023). Engineered bacteria titrate hydrogen sulfide and induce concentration-dependent effects on the host in a gut microphysiological system. Cell Rep. 42 (12), 113481. 10.1016/j.celrep.2023.113481 37980564 PMC10791167

[B17] Hu J. Fang Y. Huang X. Qiao R. Quinn J. F. Davis T. P. (2021). Engineering macromolecular nanocarriers for local delivery of gaseous signaling molecules. Adv. Drug Deliv. Rev. 179, 114005. 10.1016/j.addr.2021.114005 34687822

[B18] Ji X. Zhong Z. (2022). External stimuli-responsive gasotransmitter prodrugs: chemistry and spatiotemporal release. J. Control Release 351, 81–101. 10.1016/j.jconrel.2022.09.026 36116579

[B19] Jiang L. Wu Y. Xu Z. Hou M. Chen S. Cheng C. (2024). Harnessing hydrogen sulfide in injectable hydrogels that guide the immune response and osteoclastogenesis balance for osteoporosis treatment. Mater Today Bio 29, 101338. 10.1016/j.mtbio.2024.101338 39649246 PMC11625156

[B20] Lee J. K. Kim D. S. Park S. Y. Baek S. W. Jung J. W. Kim T. H. (2023). Nitric oxide-releasing bioinspired scaffold for exquisite regeneration of osteoporotic bone via regulation of homeostasis. Adv. Sci. (Weinh) 10 (6), e2205336. 10.1002/advs.202205336 36581472 PMC9951336

[B21] Li J. Han Q. Chen H. Liu T. Song J. Hou M. (2022). Carbon monoxide-releasing Molecule-3 enhances osteogenic differentiation of rat bone marrow mesenchymal stem cells via miR-195-5p/Wnt3a pathway. Drug Des. Devel Ther. 16, 2101–2117. 10.2147/dddt.S367277 35812136 PMC9259429

[B22] Liu C. Chen Y. Wang Y. Wang D. Sun J. Sun J. (2025). Metal-phenolic encapsulation of carbon monoxide releasing molecule for enhanced gas therapy of periodontitis. Mater Today Bio 34, 102213. 10.1016/j.mtbio.2025.102213 40893368 PMC12396554

[B23] Mancini L. Moradi-Bidhendi N. Becherini L. Martineti V. MacIntyre I. (2000). The biphasic effects of nitric oxide in primary rat osteoblasts are cGMP dependent. Biochem. Biophys. Res. Commun. 274 (2), 477–481. 10.1006/bbrc.2000.3164 10913363

[B24] Maruyama M. Rhee C. Utsunomiya T. Zhang N. Ueno M. Yao Z. (2020). Modulation of the inflammatory response and bone healing. Front. Endocrinol. (Lausanne) 11, 386. 10.3389/fendo.2020.00386 32655495 PMC7325942

[B25] Ortega J. S. Corrales-Orovio R. Ralph P. Egaña J. T. Gentile C. (2023). Photosynthetic microorganisms for the oxygenation of advanced 3D bioprinted tissues. Acta Biomater. 165, 180–196. 10.1016/j.actbio.2022.05.009 35562006

[B26] Otsuka E. Hirano K. Matsushita S. Inoue A. Shigehisa H. Yamaguchi A. (1998). Effects of nitric oxide from exogenous nitric oxide donors on osteoblastic metabolism. Eur. J. Pharmacol. 349 (2), 345–350. 10.1016/S0014-2999(98)00190-3 9671116

[B27] Oza P. P. Kashfi K. (2023). The triple crown: NO, CO, and H(2)S in cancer cell biology. Pharmacol. Ther. 249, 108502. 10.1016/j.pharmthera.2023.108502 37517510 PMC10529678

[B28] Pan P. Liu T. Zhang L. Zhang X. Z. (2026). Living materials for gas therapy. Adv. Drug Deliv. Rev. 228, 115738. 10.1016/j.addr.2025.115738 41248756

[B29] Percival K. M. Paul V. Husseini G. A. (2024). Recent advancements in bone tissue engineering: integrating smart scaffold technologies and bio-responsive systems for enhanced regeneration. Int. J. Mol. Sci. 25 (11), 6012. 10.3390/ijms25116012 38892199 PMC11172494

[B30] Robert B. Subramaniam S. (2022). Gasotransmitter-induced therapeutic angiogenesis: a biomaterial prospective. ACS Omega 7 (50), 45849–45866. 10.1021/acsomega.2c05599 36570231 PMC9773187

[B31] Rong F. Wang T. Zhou Q. Peng H. Yang J. Fan Q. (2023). Intelligent polymeric hydrogen sulfide delivery systems for therapeutic applications. Bioact. Mater 19, 198–216. 10.1016/j.bioactmat.2022.03.043 35510171 PMC9034248

[B32] Ryter S. W. (2019). Heme oxygenase-1/carbon monoxide as modulators of autophagy and inflammation. Arch. Biochem. Biophys. 678, 108186. 10.1016/j.abb.2019.108186 31704095

[B33] Sarkar S. Kumar R. Matson J. B. (2024). Hydrogels for gasotransmitter delivery: nitric oxide, carbon monoxide, and hydrogen sulfide. Macromol. Biosci. 24 (1), e2300138. 10.1002/mabi.202300138 37326828 PMC11180494

[B34] Sun H. Xu J. Wang Y. Shen S. Xu X. Zhang L. (2023). Bone microenvironment regulative hydrogels with ROS scavenging and prolonged oxygen-generating for enhancing bone repair. Bioact. Mater 24, 477–496. 10.1016/j.bioactmat.2022.12.021 36714330 PMC9843284

[B35] Sun J. Wang W. Hu X. Zhang X. Zhu C. Hu J. (2023). Local delivery of gaseous signaling molecules for orthopedic disease therapy. J. Nanobiotechnology 21 (1), 58. 10.1186/s12951-023-01813-6 36810201 PMC9942085

[B36] Suvarnapathaki S. Wu X. Zhang T. Nguyen M. A. Goulopoulos A. A. Wu B. (2022). Oxygen generating scaffolds regenerate critical size bone defects. Bioact. Mater 13, 64–81. 10.1016/j.bioactmat.2021.11.002 35224292 PMC8843972

[B37] Tsukasaki M. Takayanagi H. (2019). Osteoimmunology: evolving concepts in bone-immune interactions in health and disease. Nat. Rev. Immunol. 19 (10), 626–642. 10.1038/s41577-019-0178-8 31186549

[B38] Wan M. Chen H. Wang Q. Niu Q. Xu P. Yu Y. (2019). Bio-inspired nitric-oxide-driven nanomotor. Nat. Commun. 10 (1), 966. 10.1038/s41467-019-08670-8 30814497 PMC6393443

[B39] Wang W. Yeung K. W. K. (2017). Bone grafts and biomaterials substitutes for bone defect repair: a review. Bioact. Mater 2 (4), 224–247. 10.1016/j.bioactmat.2017.05.007 29744432 PMC5935655

[B40] Wang X. Yang C. Yu Y. Zhao Y. (2022). *In situ* 3D bioprinting living photosynthetic scaffolds for autotrophic wound healing. Res. (Wash D C) 2022, 9794745. 10.34133/2022/9794745 35387266 PMC8961369

[B41] Wang Z. Jin A. Yang Z. Huang W. (2023). Advanced nitric oxide generating nanomedicine for therapeutic applications. ACS Nano 17 (10), 8935–8965. 10.1021/acsnano.3c02303 37126728 PMC10395262

[B42] Wang Z. Zhang D. Jian C. Qi B. Li Z. Yu A. (2025). Pathological bone regeneration in soft tissues: a narrative review of traumatic heterotopic ossification. Regen. Med. Rep. 2 (3), 130–136. 10.4103/REGENMED.REGENMED-D-24-00023

[B43] Wei H. Cui J. Lin K. Xie J. Wang X. (2022). Recent advances in smart stimuli-responsive biomaterials for bone therapeutics and regeneration. Bone Res. 10 (1), 17. 10.1038/s41413-021-00180-y 35197462 PMC8866424

[B44] Won J. E. Kim W. J. Shim J. S. Ryu J. J. (2022). Guided bone regeneration with a nitric-oxide releasing polymer inducing angiogenesis and osteogenesis in critical-sized bone defects. Macromol. Biosci. 22 (10), e2200162. 10.1002/mabi.202200162 35895972

[B45] Zafonte R. D. Wang L. Arbelaez C. A. Dennison R. Teng Y. D. (2022). Medical gas therapy for tissue, organ, and CNS protection: a systematic review of effects, mechanisms, and challenges. Adv. Sci. (Weinh) 9 (13), e2104136. 10.1002/advs.202104136 35243825 PMC9069381

[B46] Zhao M. Ko S.-Y. Garrett I. R. Mundy G. R. Gutierrez G. E. Edwards J. R. (2018). The polyphenol resveratrol promotes skeletal growth in mice through a sirtuin 1-bone morphogenic protein 2 longevity axis. Br. J. Pharmacol. 175 (21), 4183–4192. 10.1111/bph.14477 30125963 PMC6177622

[B47] Zheng J. Wang W. Gao X. Zhao S. Chen W. Li J. (2022). Cascade catalytically released nitric oxide-driven nanomotor with enhanced penetration for antibiofilm. Small 18 (52), e2205252. 10.1002/smll.202205252 36344450

[B48] Zhou T. Liu W. Lai H. Liu Y. Su W. Xu Z. (2023). Hydrogen sulfide promotes osteogenesis by modulating macrophage polarization. Int. Immunopharmacol. 115, 109564. 10.1016/j.intimp.2022.109564 36571916

[B49] Zhou Y.K Y. K. Han C. S. Zhu Z. L. Chen P. Wang Y. M. Lin S. (2024). M2 exosomes modified by hydrogen sulfide promoted bone regeneration by moesin mediated endocytosis. Bioact. Mater 31, 192–205. 10.1016/j.bioactmat.2023.08.006 37593496 PMC10429289

[B50] Zhou Z. Liu Y. Li W. Zhao Z. Xia X. Liu J. (2024). A self-adaptive biomimetic periosteum employing nitric oxide release for augmenting angiogenesis in bone defect regeneration. Adv. Healthc. Mater 13 (3), e2302153. 10.1002/adhm.202302153 37922941

