# Supplementary material for: Bioactive small-molecule gas delivery for enhanced bone regeneration: modulation of cellular signaling and remodeling of the regenerative microenvironment
Source: Front Cell Dev Biol. 2026 Oct 12;14:1992564. doi: 10.3389/fcell.2026.1992564 (PMC13696028; doi:10.3389/fcell.2026.1992564)
Supplement: Supplementary file 1 [file Supplementaryfile1.docx]

Supplementary Material

# 1 Supplementary Figures

**Figure 1.**  Schematic overview of bioactive small-molecule gas-delivering biomaterials and their regulatory functions in bone regeneration.

**Figure 2.**  Gasotransmitter-releasing biomaterials for enhanced bone regeneration: from gas donor incorporation to biological regulation and tissue repair.
